# MYNAH Registry: A novel approach to decoding the natural history of degenerative cervical myelopathy

**DOI:** 10.1016/j.jcot.2025.103075

**Published:** 2025-05-26

**Authors:** Nashwa Najib, Alisha W. Sial, Hussain Bohra, Ashish D. Diwan

**Affiliations:** a Spine Labs & Spine Service, Department of Orthopaedic Surgery, Discipline of Surgery, St George Hospital, Sydney, Australia; b St George & Sutherland Clinical Campus, School of Clinical Medicine, UNSW Medicine & Health, University of New South Wales (UNSW), Sydney, Australia; c Department of Orthopaedic Surgery, Adelaide Medical School, Faculty of Health and Medical Sciences, University of Adelaide, Australia

## Abstract

**Figure:**
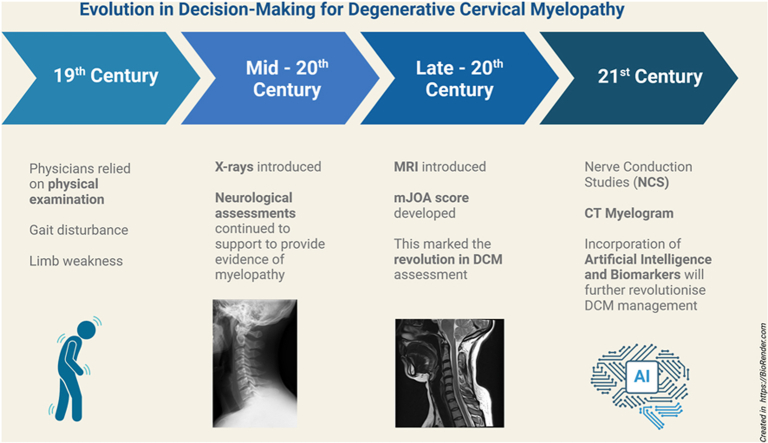

## 1 Introduction

Degenerative Cervical Myelopathy (DCM) is a progressive neurological disorder resulting from cervical spine degeneration and spinal cord compression. Clinical presentation ranges from asymptomatic cases to complete paralysis, with symptoms including neck pain, upper and lower limb weakness or numbness, loss of manual dexterity, gait imbalance, and bladder or bowel dysfunction, contributing to a stepwise decline in quality of life.^1^, ^2^, ^3^ Common physical signs include hyperreflexia, clonus, Hoffmann's sign, finger escape sign, extensor plantar response, spasticity, unstable gait, and sensory deficits.^1^, ^2^, ^3^

Historically, grouped under the term Cervical Spondylotic Myelopathy (CSM), DCM has since been recognised as a distinct clinical entity. By 2009, it was acknowledged as a research priority due to its severe prognosis if left undiagnosed or untreated.^4^^,^^5^ Recent findings, such as the association between intramedullary signal intensity (ISI) and non-spinal degeneration^6^ have further advanced the understanding of DCM, highlighting its impact on the aging population and its influence on functional independence.

The shift in focus has been catalysed by initiatives like the AO Spine RECODE-DCM project, which established the top 10 research priorities for DCM, emphasising the urgent need to understand its natural history through longitudinal studies.^7^ Such studies provide critical insights into disease progression, variability and prognostic factors, informing clinical decision-making.

Patient Registries and Databases have become essential tools for capturing longitudinal data. With the evolution of electronic health records (EHR) from the 1960s to the 1990s,^8^ registries now enable systematic tracking of disease trajectories and treatment outcomes. In DCM, these data are crucial for identifying disease patterns, optimising management strategies, and guiding future research.

Advances in diagnostic technologies, artificial intelligence, and multi-omics research are further transforming the landscape, offering new opportunities to understand DCM at structural, cellular, and molecular levels.^9^ Continuous innovation will be pivotal in refining diagnostic criteria, prognostication, and therapeutic approaches for this complex condition.

A novel approach to understanding the natural history of DCM, its biological basis, assessment and monitoring was established through the MYelopathy NAtural History (MYNAH) Registry. Establishment of the MYNAH Registry is a small step; however, it marks a giant leap for researchers addressing the RECODE-DCM research priorities. We present the existing challenges in the diagnosis of DCM and provide an overview of the inception and implementation of the MYNAH Registry, highlighting its vital importance as an adaptive platform in improving our understanding and clinical decision-making in the management of DCM.

## 2 Evolution of the MYNAH registry

The **MY**elopathy **NA**tural **H**istory (MYNAH) (UNSW HREC iRECS3634) Registry was established in Australia in December 2022, representing the first global effort to systematically study the natural history of DCM. Listed on the Australian Register of Clinical Registries (ACSQHC-ARCR-258), MYNAH is an opt-in, investigator-initiated, multicenter, prospective, longitudinal study recruiting both operated and non-operated DCM patients across Australia.

Co-led by Dr Ashish D Diwan and Dr Nashwa Najib, the registry captures Practitioner-Reported Outcome Measures (PrROMs) such as modified Japanese Orthopaedic Association (mJOA) Score and Nurick Grade; alongside Patient-Reported Outcome Measures (PROMs) including the Neck Disability Index (NDI), EQ-5D-5L and EQ-VAS with six-monthly follow-up intervals. A detailed description of the registry design, methodology and baseline characteristics of participants has been published.^10^ The MYNAH Registry aggregates comprehensive clinical, imaging, treatment, and outcome data, enabling large-scale analyses to evaluate prognoses, quality of life outcomes and treatment effectiveness. It facilitates comparative effectiveness research and supports the development of personalised care strategies by identifying patient subgroups with differing treatment responses.

**Fig. 1:**
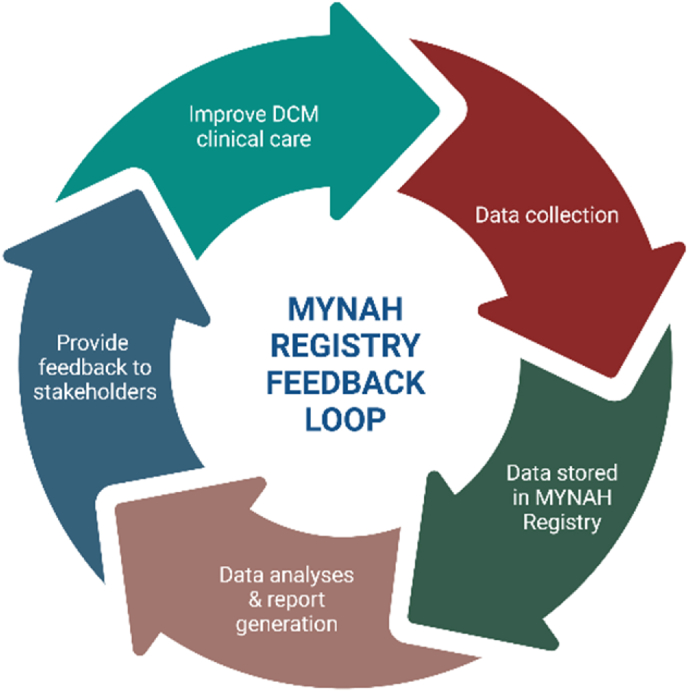
MYNAH registry feedback loop (created in https://BioRender.com).

Importantly, MYNAH operates under the Australian Clinical Quality Registries (CQR) Feedback Loop model,^11^ converting collected data into actionable insights that inform quality improvement initiatives and enhance DCM care nationally. While still in early stages, MYNAH has already yielded critical learnings regarding symptom specificity, clinical assessment, and management patterns that will inform future longitudinal studies (Fig. 1).

## 3 Headache as a clinical feature in DCM

The clinical presentation of Degenerative Cervical Myelopathy (DCM) is heterogeneous, involving motor, sensory, autonomic, and pain-related impairments. While neck pain is a well-recognised hallmark of DCM, headache remains an underappreciated symptom. Emerging evidence suggests that cervicogenic headache originating from structural or functional abnormalities of the cervical spine may form part of the DCM symptom complex.^12^ Degenerative changes such as disc degeneration, osteophytosis, and ligamentous hypertrophy can irritate upper cervical neural structures, particularly the C1–C3 roots and the trigeminocervical complex (TCC).^13^ In addition, spinal cord ischemia and chronic neuroinflammation, central to DCM pathophysiology may sensitise central pain pathways, further contributing to headache development. Dysregulation of inflammatory cytokines, including interleukin-6 (IL-6) and tumor necrosis factor-alpha (TNF-α), provides additional mechanistic links between DCM and headache syndromes.^14^ Although headache is not currently included in formal DCM diagnostic criteria, its recognition particularly in patients with chronic neck pain and subtle neurological signs could support earlier diagnosis and intervention, potentially preventing irreversible spinal cord injury.

## 4 Symptomatology of DCM

Alongside clinical examination, symptom assessment is essential for early recognition and monitoring of DCM, which presents with a broad spectrum of sensory, motor, and autonomic dysfunctions (Table 1).

**Table 1:** Common symptoms associated with DCM providing descriptions of symptom presentation and their clinical relevance. These symptoms reflect the diverse sensory, motor, and autonomic impairments characteristic of DCM.

Symptom	Description	Clinical relevance
Neck Pain	Persistent or episodic cervical discomfort, often movement aggravated	Early indicator of cervical spinal degeneration
Autonomic Dysfunction	Urinary urgency, incontinence, or retention.	Spinal cord involvement in autonomic pathways
Hand Numbness	Loss of sensation or tingling in one or both hands.	Dorsal column dysfunction/nerve root compression
Hand Paresthesia	Abnormal tingling or prickling sensations	Spinal cord involvement
Upper Extremity Weakness	Progressive loss of strength in one or both upper limbs.	Affects fine motor control and grip strength
Loss of Dexterity	Difficulty with fine motor tasks (e.g., buttoning shirts or writing)	Hand dysfunction in DCM
Gait Instability	Frequent stumbling, poor balance, or unexplained falls.	Spinal cord-mediated proprioceptive impairment
Lhermittes's Phenomenon	Electric shock-like sensation radiating down the spine and limbs upon neck flexion.	Dorsal column dysfunction
Upper Extremity Tremor	Involuntary rhythmic muscle contractions, exacerbated by movement.	Corticospinal tract dysfunction
Vertigo	Dizziness or spinning sensation with neck movements.	Compromised proprioceptive signaling from cervical mechanoreceptors
Hypalgesia	Reduced pain sensitivity (e.g.,diminished response to pinprick testing)	Dorsal column impairment
Jitteriness	Subjective unsteadiness or shakiness.	Indicative of spinal cord dysfunction
Apraxia	Difficulty coordinating voluntary movements despite intact strength	Corticospinal dysfunction

The clinical course of DCM often follows an 'inverted U′ trajectory, with gradual symptom worsening, a temporary plateau, and further decline if untreated. This reflects chronic spinal cord compression, ischemia, and progressive axonal injury. Timely diagnosis and early surgical decompression are critical to halt deterioration and prevent irreversible functional loss.

## 5 Clinical examination in the diagnosis of DCM

Clinical examination remains pivotal for the early diagnosis and monitoring of DCM. Hyperreflexia, upper motor neuron signs, and gait instability are key indicators of spinal cord compression, while progressive sensorimotor and autonomic dysfunctions warrant urgent imaging and specialist referral (Table 2).

**Table 2:** Key diagnostic clinical signs for DCM detailing the examination methods, positive findings, and the clinical significance of each sign in identifying spinal cord compression and neurological dysfunction. These signs play a critical role in the early detection and monitoring of DCM progression.

Test	Method	Positive finding	Clinical significance
Tromner's sign	Tap palmar side of stabilized middle finger	Thumb or index hyperreflexia	Increased corticospinal excitability
Babinski's sign	Stroke lateral plantar foot	Great toe dorsiflexion, toe fanning	Upper motor neuron dysfunction
Hoffmann's sign	Flick distal phalanx of middle finger	Thumb and index finger flexion	Spinal cord hyperexcitability
Clonus	Rapid dorsiflexion of ankle	≥3 rhythmic beats	Corticospinal tract involvement
Inverted supinator sign	Tap brachioradialis tendon	Finger flexion or elbow extension	Cervical spinal cord dysfunction
Rhomberg's sign	Patient stands with feet together, eyes closed	Loss of balance	Proprioceptive dysfunction (posterior column impairment)
Finger Escape sign	Hands palm-down, fingers extended	Abduction of fifth finger	Intrinsic hand weakness (myelopathy)
Grip Release test	Rapid grip and release cycles for 10 s	Failure to complete ≥20 cycles	Myelopathic hand dysfunction
Hyperreflexia at Brachioradialis	Strike lower radius	Exaggerated supination	Upper motor neuron involvement
Hyperreflexia at Biceps or Triceps	Strike tendon (biceps/triceps)	Exaggerated elbow flexion/extension	Corticospinal hyperexcitability
Hyperreflexia at Patella	Strike patellar tendon	Brisk knee extension	Upper motor neuron dysfunction
Gait Analysis	Gait Analysis	Spasticity, ataxia, wide-based stance	Significant spinal cord compression

The systematic review and meta-analysis by Jiang et al.^15^ demonstrated that Tromner's sign and hyperreflexia are the most sensitive indicators of DCM, with Tromner's sign also showing high specificity, making it a valuable diagnostic tool. Babinski's sign, clonus, and the inverted supinator sign exhibited near-perfect specificity, supporting their use as confirmatory signs despite lower sensitivity. However, signs such as Hoffmann's sign, Babinski's sign, and hyperreflexia, while diagnostically useful, do not consistently correlate with disease severity, limiting their utility in monitoring progression.

Although DCM is a clinical-radiological diagnosis, significant gaps persist in defining the most predictive clinical features. A recent scoping review^16^ identified key diagnostic signs with the highest predictive value. Standardising clinical criteria, akin to predictive tools like the Wells’ Score, could enhance early recognition, especially in primary care settings. With a rising DCM prevalence in the aging population, the development of validated diagnostic frameworks is critical to improving patient outcomes.

## 6 Insights from tracking natural history through the MYNAH registry

The MYNAH Registry has rapidly established itself as a valuable tool for studying the natural history of DCM. Over two years, more than 70 patients have been recruited from diverse neurosurgical and spine centres across Australia, capturing a broad spectrum of age ranges and geographical backgrounds. Reflecting real-world clinical practice, enrolment criteria are intentionally broad, allowing any patient diagnosed with DCM by a spine surgeon or neurosurgeon to participate, thus embracing the heterogeneity inherent in DCM diagnosis and management.

To ensure data quality, a comprehensive data dictionary has been developed. Rigorous governance measures including bi-annual audits, annual compliance checks, and real-time data monitoring using a traffic light system have been instituted to maintain consistency, accuracy, and adherence to standardised operating procedures across sites.

## 7 Case showing how registry monitoring changes the operative approach

**Fig. 2:**
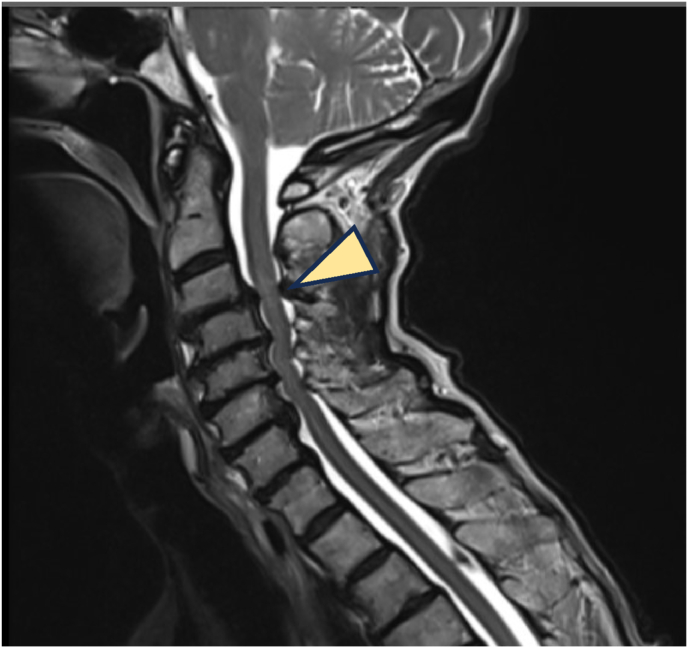
T2W MRI of the cervical spine revealed multi-level compression C3-7 and cord signal change most pronounced at C3-4 (arrowhead). After posterior cervical decompression and fusion, she showed significant improvement in arm strength and pain.

A 77-year-old female with a 20-year history of neck and bilateral arm pain (VAS 8/10) impacting daily activities was initially managed non-operatively for multilevel C3–7 compression without cord signal change (Fig. 2). She had a history of lumbar decompression and a positive family history of DCM. At 2-year follow-up, symptoms worsened (neck VAS 9/10, arm VAS 10/10, NDI 72 %, EQ-5D 0.062) with new cord signal changes maximal at C3–4. Following multidisciplinary consensus, she underwent C2–T2 posterior cervical decompression and fusion. Postoperatively, she developed DVT, managed conservatively. At 6 weeks, she showed clinical improvement; by 6 months, EQ-5D improved to 0.234 and NDI to 48 %. She developed hypertrophic bony remodeling without neurological deficit. At 8 months, revision surgery for wound dehiscence was performed with good recovery. At final follow-up, she achieved significant functional improvement (NDI 46 %, EQ-5D 0.707, Nurick Grade 0, mJOA 12). Despite initial absence of cord signal change, surgical intervention was appropriate given her progressive functional decline and was supported by spine specialist consensus.

## 8 Case of cord signal changes following Anterior Cervical Decompression and Fusion (ACDF)

**Fig. 3:**
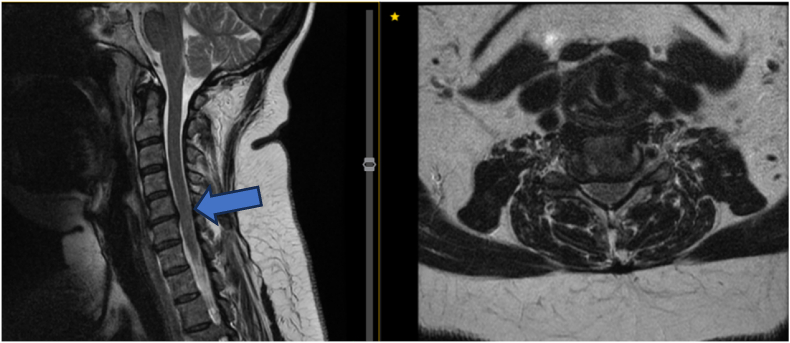
MRI revealed chronic contained C5-6 disc herniation with loss of disc height and bilateral foraminal stenosis.

**Fig. 4:**
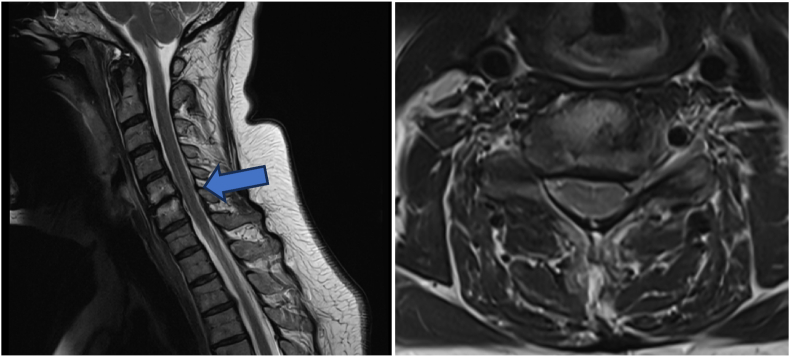
MRI at one-year post-op revealed a very subtle cord signal change (arrowhead). A T2W axial cut at the level of C5-6 shown on the right.

A 58-year-old female presented with 3.5 years of neck and left arm pain following a workplace incident. Neurological exam was normal. MRI showed chronic contained C5–6 disc herniation, disc height loss, and bilateral foraminal stenosis; initial management was conservative (Fig. 3). Due to persistent arm pain, she underwent C5–6 Anterior Cervical Decompression and Fusion (ACDF). Post-op, she reported moderate symptom relief but fluctuating NDI scores. At 6 and 9 months, she showed improved neck symptoms and functional recovery (NDI 34 %, EQ-5D 0.535, mJOA 18), despite subtle gait disturbance and positive Rhomberg. At 1-year follow-up, MRI revealed subtle cord signal changes without major clinical deterioration (mJOA 14, moderate myelopathy) (Fig. 4). She remains clinically stable under close monitoring. The cord changes are likely related to underlying degenerative processes rather than surgical injury.

## 9 Case of degenerative cervical myelopathy (DCM) and positive family history of DCM

**Fig. 5:**
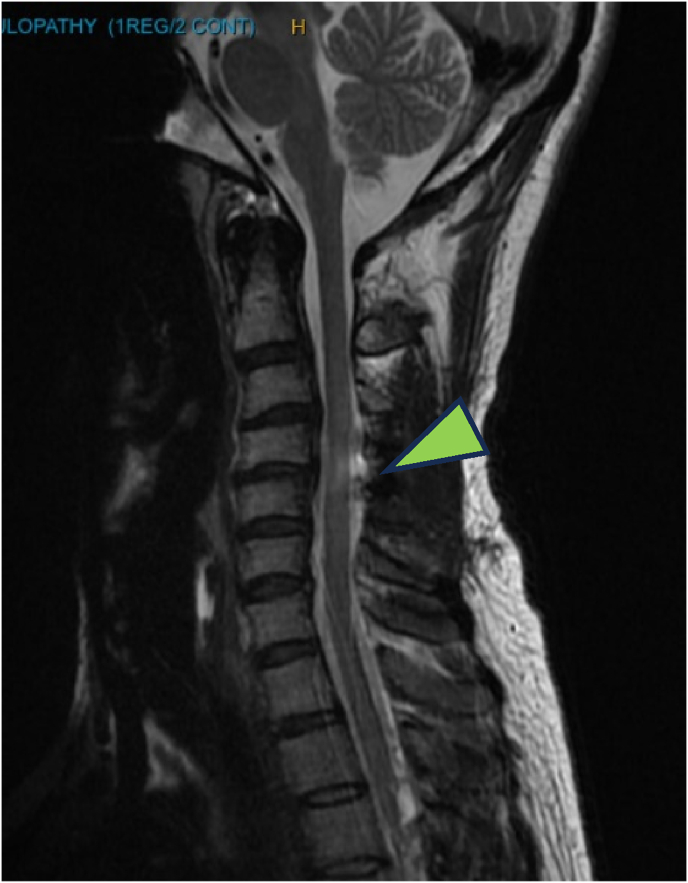
A 42-year-old male presented with over 10 yr history of neck pain and arm pain. MRI showed C4-5 disc herniation. (arrowhead).

A 42-year-old male with over 10 years of neck and arm pain (VAS 7/10, EQ-5D 0.611, NDI 48 %) and a positive first-degree family history of DCM presented for surgical opinion. Examination revealed global hyperreflexia, bilateral positive Hoffmann's sign (right dominant), suppressed biceps reflexes, and preserved motor strength (mJOA 15). MRI showed a contained C4–5 disc herniation with a 1.6 cm cord signal change (Fig. 5). He was advised close monitoring and avoidance of contact sports. Following a diving injury causing transient quadriparesis, he recovered with residual arm pain. Repeat MRI showed stable cord signal change and left foraminal stenosis. Ongoing six-monthly monitoring was recommended given his genetic risk and clinical course. This case highlights the importance of recognising family history and genetic predisposition in DCM diagnosis and management. Incorporating familial risk into clinical assessment supports early detection and tailored monitoring. Regular neurological evaluations are essential to identify progression early and allow timely intervention, improving outcomes and preserving function. The heterogeneous nature of DCM necessitates individualised management. Surgical intervention remains the definitive treatment for moderate-to-severe DCM, with options including Anterior Cervical Discectomy and Fusion (ACDF), Posterior Cervical Decompression and Fusion (PCDF), laminectomy, laminoplasty, and cervical disc arthroplasty. The choice of approach depends on compression location, cervical alignment, comorbidities and surgeon preference resulting in variability in practice.

## 10 Variability of surgical approaches and implications for decision-making

Of the 60 patients recruited in the MYNAH Registry, 24 patients had surgical status (+) at the time of recruitment, i.e. these patients had undergone C-spine surgery before recruitment in the registry. Of these 24 patients, 13 (54.2 %) had ACDF, 4 (16.6 %) PCDF, 2 (8.3 %) Decompression alone, 2 (8.3 %) Laminectomy, 1 (4.2 %) Laminoplasty, 1 (4.2 %) Cervical Discectomy, and 1 (4.2 %) vertebral alignment. ACDF emerged as the most commonly employed technique. This diversity in practice contrasts with patterns observed in Japan, where a higher prevalence of DCM – partly attributed to the increased incidence of OPLL^17^ – and comprehensive healthcare coverage contribute to a greater frequency of cervical decompression surgeries.^18^(27).

The choice of surgical approach in DCM is inherently individualised and reflects a complex interplay of clinical and anatomical factors. These include the location and extent of spinal cord compression, cervical alignment, patient comorbidities, and surgeon experience. Anterior, posterior, or combined approaches may be selected based on these considerations, with no single technique universally applicable across all cases. A randomized controlled trial by Ghogawala et al. demonstrated that dorsal approaches resulted in superior patient-reported functional outcomes compared to ventral approaches at one year, underscoring the importance of tailoring surgical strategy to the patient's specific pathology.^19^

Despite these advances, current DCM management guidelines remain limited and imprecise, reflecting substantial knowledge gaps, particularly regarding the natural history of the disease.^20^ The heterogeneity of DCM presentations, subjective nature of severity assessments (e.g., mJOA Score, Nurick Grade), and variability in clinical progression continue to challenge standardisation. Consequently, close clinical monitoring and regular follow-up are essential to detect early neurological deterioration and to inform timely surgical intervention. Understanding the variability in surgical management is critical to optimising outcomes and highlights the need for further research to refine evidence-based guidelines.

Early observations from the MYNAH Registry underscore the significant variability in DCM progression, mirroring the clinical heterogeneity of the condition. As diagnostic capabilities evolve, future classifications may incorporate emerging biomarkers and advanced imaging techniques, enabling more precise disease definitions. The identification of distinct DCM subgroups based on consistent clinical or molecular hallmarks is also anticipated, offering opportunities for more individualised care.

Systematic, longitudinal tracking of DCM through patient registries like MYNAH is critical given the variability in surgical decision-making and ongoing gaps in clinical consensus. By continuously capturing and analysing real-world data, MYNAH aims to refine the understanding of DCM, supporting the development of more accurate diagnostic criteria and personalised management strategies to optimise patient outcomes.

## 11 Future utilisation of the MYNAH registry

The MYNAH Registry Biobank stores participant plasma and serum aliquots at −80 °C in secure, access-restricted Spine Lab facilities. Specimen management is streamlined using OpenSpecimen. A nested DCM Blood Biomarker Study, employing global and targeted proteomics, is underway to enhance understanding of DCM's natural history. Phase I (Biomarker Discovery) was completed in early 2025, with Phase II (Verification) beginning last quarter of 2025, and Phase III (Validation) scheduled for late 2026.

The MYNAH Registry provides an adaptive platform for longitudinal observational and biomarker studies. Insights generated will advance knowledge of DCM progression and facilitate the development of clinical guidelines, enabling more personalised treatment strategies and improving patient outcomes.

## CRediT authorship contribution statement

**Nashwa Najib:** Co-chief investigator, Conceptualization, Methodology, Software, Formal analysis, Investigation, Data curation, Writing – original draft, Writing – review & editing, Visualisation, Project administration, and Funding acquisition. **Alisha W. Sial:** Data curation, Writing – original draft, and, Writing – review & editing. **Hussain Bohra:** Data curation, Writing – original draft, and, Writing – review & editing. **Ashish D. Diwan:** Chief Investigator, Conceptualization, Methodology, Validation, Investigation, Resources, Data curation, Writing – review & editing, Visualisation, Supervision, and Funding acquisition.

## Informed consent

Informed consent has been obtained from patients for the study participation and publication.

## Ethics number

University of New South Wales (UNSW) Human Research Ethics Committee (HREC) Executive (iRECS3634).

## Funding statement

The MYNAH Registry is partly funded by the 10.13039/100015069South Eastern Sydney Local Health District (10.13039/100015069SESLHD) RES-ON Grant Round 2, November 2023.

## Declaration of competing interest

None.
